# Mechanism of Nardostachyos Radix et Rhizoma–Salidroside in the treatment of premature ventricular beats based on network pharmacology and molecular docking

**DOI:** 10.1038/s41598-023-48277-0

**Published:** 2023-11-25

**Authors:** Liu Shuyuan, Chen Haoyu

**Affiliations:** 1 The First Clinical Medical School, Shandong University of Traditional Chinese Medicine, Jinan, ShanDong People’s Republic of China 250013; 2 Cardiology, Affiliated Hospital of Shandong University of Traditional Chinese Medicine, Jinan, ShanDong People’s Republic of China 250011

**Keywords:** Biological techniques, Cell biology, Drug discovery, Molecular biology, Cardiology, Molecular medicine, Pathogenesis

## Abstract

To analyse the mechanism of Nardostachyos Radix et Rhizoma–Salidroside in the treatment of Premature Ventricular Brats by using network pharmacology and molecular docking and to provide the basis for developing the use of experimental and clinical traditional Chinese medicine. The chemical compositions of Nardostachyos Radix et Rhizoma and Salidroside were determined, and their related targets were predicted. The disease-related targets were obtained by searching the common disease databases Genecards, OMIM, Drugbank and DisGeNET, and the intersection between the predicted targets and the disease targets was determined. Then using the STRING database to set up the protein‒protein interactions (PPIs) network between Nardostachyos Radix et Rhizoma–Salidroside and the common targets of PVB. An “herb-ingredient-target” network was constructed and analyzed by Cytoscape3.7.2 software. Using the metascape database to analysis the predicted therapeutic targets based on the GO and KEGG. Finally, molecular docking technology was used toconfirm the capacity of the primary active ingredients of the 2 herbs to bind to central targets using the online CB-Dock2 database. 41 active components of Nardostachyos Radix et Rhizoma–Salidroside were detected, with 420 potential targets of action, with a total of 1688 PVB targets, and the top 10 core targets of herb-disease degree values were AKT1, TNF, GAPDH, SRC, PPARG, EGFR, PTGS2, ESR1, MMP9, and STAT3. KEGG analysis indicated that its mechanism may be related to the calcium signalling pathway, cancer signalling pathway, AGE-RAGE signalling pathway and other pathways. Molecular docking suggested that main of the active ingredients of the Nardostachyos Radix et Rhizoma–Salidroside pairs were well bound to the core targets. Based on novel network pharmacology and molecular docking validation research methods, we revealed for the first time the potential mechanism of Nardostachyos Radix et Rhizoma–Salidroside in PVB therapy.

## Introduction

Premature ventricular beats (PVB), also known as ventricular presystole, is a common clinical arrhythmia that refers to spontaneous excitation of the ventricle that is not receiving signals from the sinus node, resulting in premature ventricular depolarisation. Clinically, some patients may have no obvious symptoms, while others may experience palpitations, dizziness, fatigue, chest tightness, etc.^1^. Traditional Chinese medicine (TCM) puts PVB into the scope of "palpitations", which started from "Yellow Emperor's Classic of Internal Medicine", and its disease is located in the heart. According to Chinese traditional medicine, the main pathological mechanisms are exogenous disease, injury due to diet, internal injury due to emotional disorder, physical deficiency and prolonged illness. The disease mechanism is mostly based on the deficiency of qi blood yin and yang of the original organism, followed by blood stasis and phlegm. At present, modern medical treatment mainly includes anti-arrhythmic drugs and surgery, which are effective but easily cause liver and kidney function damage in the long term, require higher costs and patient compliance is not high. In recent years, Chinese medicine has gradually embodied unique advantages in the treatment of PVB^2^.

According to modern pharmacological findings^3^, the main compounds in Nardostachyos Radix et Rhizoma (NRER) are terpenoids, volatile oils, flavonoids, coumarins, lignans, sugars, etc., and NRER neophytum can affect the cellular cAMP-PKA conduction pathway, inhibit calcium overload, and promote the restoration of cardiac function^4^; it can also inhibit cardiac hypertrophy by inhibiting the expansion of the cell surface area induced by angiotensin II (AngII)^5^. Salidroside(SAL) mainly contains ketones, phenylalkyl glycosides, coumarins, organic acids, polysaccharides, amino acids, vitamins, etc.^6^, which can activate voltage-gated potassium channel proteins and calcium channel proteins to stabilise ion channels on the cell membrane, alleviate myocardial fibrosis and eliminate ectopic rhythms^7^. Other study have shown that SAL has inhibitory effects on tumor cells^8^. The treatment of diseases by Chinese medicine compounds involves multitarget and multicomponent integrated regulation, and the construction of a complex network of hurbs and disease genes and proteins through network pharmacology is conducive to the development of new drugs and provides new ideas for the subsequent treatment of diseases^9^. At present, the mechanism of NRER-SAL action is still unknown, and there have not been studied in the treatment of PVB.

Network pharmacology is a new discipline based on systems biology theory, biological system network analysis and the selection of specific signal nodes for multi-target drug molecule design. TCM compounds have the characteristics of multiple targets and levels. This mechanism is similar to the completeness, systematization and comprehensiveness of network pharmacology, so network pharmacology is suitable for studying the pharmacological mechanism of Chinese medicine compound compounds^10^. Discovering TCM from the system point of view and molecular level, the research range from "one target, one drug" to the new "network target, multi-component" model has established a new approach to TCM network pharmacology^11^. We all know that although traditional medicines provide the basis for the development of modern medicines, they still need to undergo discovery, isolation, and mechanistic studies^12^. Therefore, the early work is also very necessary.

**Figure 1:**
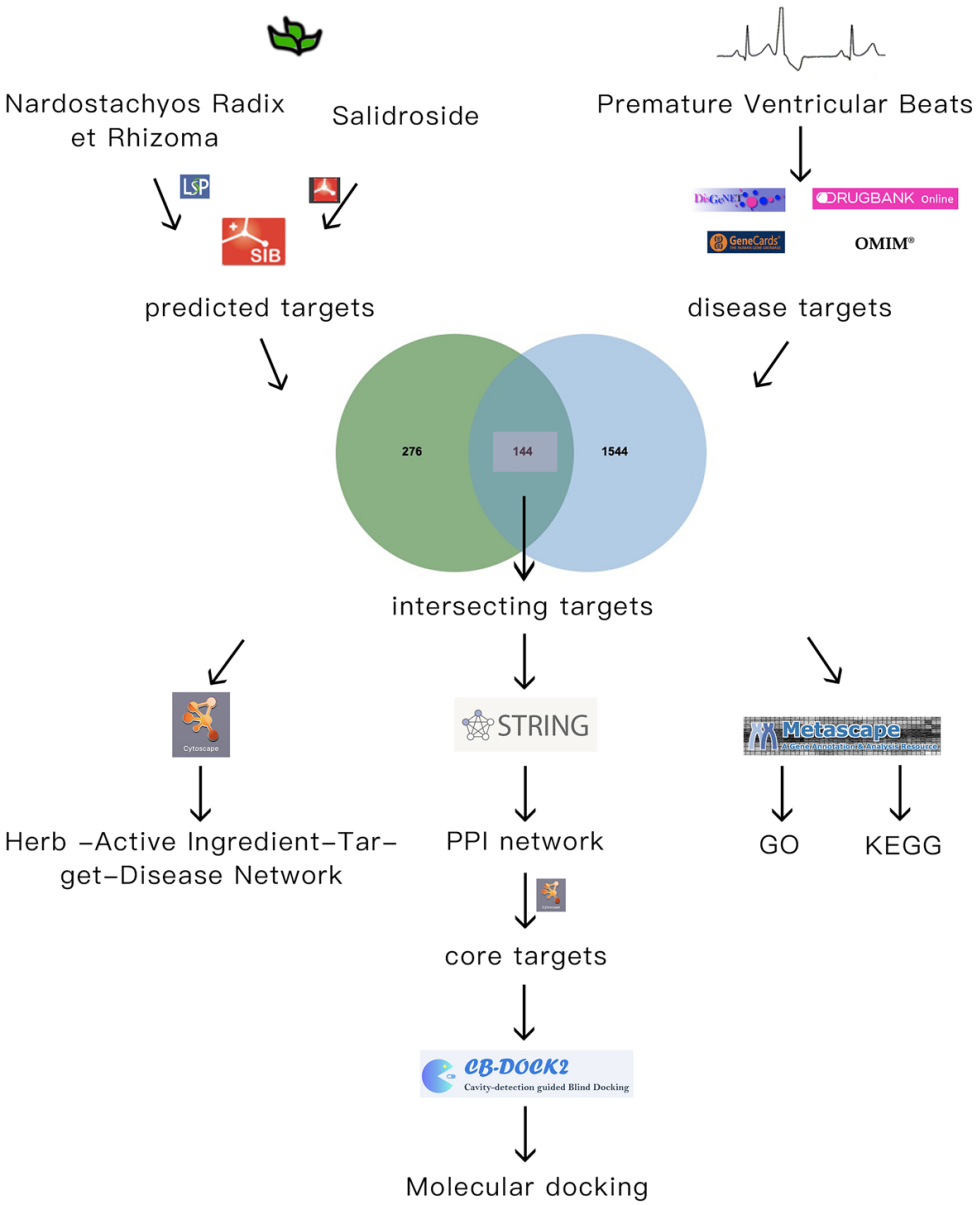
Flow chart of this study.

In this study, network pharmacology and molecular docking methods were used to investigate the key targets and mechanisms of action of NRER-SAL for the treatment of PVB, the first time to explore the possible pharmacodynamic substances and potential targets of NRER-SAL in the treatment of PVB, which will provide guidance for further experiment research, clinical research and new medicinal development in the future (Fig. 1).

## Information and methodology

### Screening of active ingredients and corresponding targets of herbs

NRER: Based on the Traditional Chinese Medicine Systems Pharmacology Database and Analysis Platform, TCMSP (http://lsp.nwu.edu.cn/tcmsp.php), a systematic pharmacological database and analytical platform database for traditional Chinese medicines and part of the literature collection at^5,5^, with two ADME attribute values of oral bioavailability (OB) ≥ 30% and drug-likeness (DL) ≥ 0.18, the active ingredient was entered into the PubChem database (https://pubchem.ncbi.nlm.nih.gov/) to record the SMILES number.

SAL: The active ingredients and chemical structures of SAL were collected through the literature^13,14^, and the canonical SMILES numbers of the active ingredients of the herb were found through PubChem and copied to the Swiss Target Prediction database (SwissADME) (http://www.swissadme.ch/). If the active ingredient was not found in PubChem, the chemical structure of the ingredient was searched through the literature and plotted in the SwissADME database, and high gastrointestinal absorption and drug-like properties (DL) ≥ 2 were taken as the screening conditions in the results and recorded.

The screened active ingredients of the above two herbal medicines were subjected to target prediction in the Swiss Target Prediction database (http://www.swisstargetprediction.ch/)^15^, and the study species was selected as "*Homo sapiens*". The study species was selected as "*Homo sapiens*", “probility > 0.1”, no target compounds were excluded, the predicted target information of the active ingredient was exported, and the gene name was recorded for follow-up work.

### Screening of targets for premature ventricular contractions

Using "premature ventricular beats" as the keyword, we screened the GeneCards database (https://www.genecards.org), Online Mendelian Inheritance in Man database (OMIM, https://omim.org/), Drugbank database (https://go.Drugbank.com/) and Disease Gene Network Database (DisGeNET, https://www.disgenet.org/) for targets of PVB and summarised and deduplicated them.

### NRER-SAL active ingredients and potential target acquisition for premature ventricular contractions

The obtained predicted targets of drug composition and the multidatabase summary targets of the disease were used to construct a Venn diagram using the Microbiotics online data analysis visualisation platform (http://www.bioinformatics.com.cn) to obtain the intersection target of the NRER-SAL pair for the treatment of PVB, which is the potential target of action of the two herbs for the treatment of the disease.

### "Herb-active ingredient-target-disease" network construction

A network diagram of "Herb-Active Ingredient-Target-Disease" network was constructed by inputting NRER-SAL pairs, their active ingredients, potential targets and PVB disease names into Cytoscape 3.7.2 software.

### PPI network construction

The intersecting targets were imported into the STRING11.0 database (https://string-db.org/cgi/input.pl), the species was selected as "*Homo sapiens*", and the interaction threshold was "highest confidence ≥ 0.4" to obtain the protein‒protein interaction network (PPI). Then the image and tsv file were saved. The results were then imported into CytoScape 3.7.2, and the software plug-in Centiscape2.2 was used to calculate the Closeness, Betweenness and Degree, where the Closeness reflects the tightness of the node to other nodes and the Betweenness indicates the degree of the node's pivotal importance in the network topology. The higher the Degree is, the higher the influence of the node in the network^16–18^, filtered and rearranged according to the degree value in ascending order.

### KEGG and GO enrichment analysis

The intersecting targets were imported into the Metascape platform^19^ (https://Metascape.org/), the species was limited to "*Homo sapiens*", and the screening condition was P < 0.01 by default. Gene Ontology (GO) functional enrichment analysis and Kyoto Gene and Genome (KEGG) pathway enrichment analysis^20–22^ were performed. GO functions included biological process (BP), cellular component (CC) and molecular function (MF), which were used to describe the target function. KEGG pathway enrichment analysis obtained the signaling pathways enriched in the core targets of PVB. The results of GO function were taken as the top 10, and the KEGG pathways were taken as the top 20 in terms of -log10 (p value) value from high to low. The results were visualised with the help of the microbiology platform and outputted in the form of bar charts and bubble charts, respectively.

### Molecular docking

The core target proteins with the top 10 degree values in the PPI network were selected for molecular docking validation with the 5 core components with the top degree values. The small molecule structures were obtained from the PubChem database, and the 3D structures of the obtained small molecules were saved in mol2 format, imported into openbabel 2.4.1 software for file conversion, and exported in pdb format. Protein structures were obtained from the PDB database (https://www.rcsb.org/) as pdb format files using the online CB-Dock2 database (http://clab.labshare.co.uk/cb-dock/php/index.php)^23,24^ for validation and visualisation analysis. In this paper, we apply a new blind docking tool called CB-Dock2, which focuses on improving docking accuracy. CB-Dock2 predicts the binding region of a given protein, calculates the center and size using a curvature based cavity detection method, and interconnects with the state-of-the-art docking software Autodock Vina. CB-Dock2 also ranks binding patterns based on Vina scores and provides an interactive 3D visualization of binding patterns. And the absolute values of the binding energies were ranked from high to low.

## Results

### Screening of active herb ingredients and disease targets

**Figure 2:**
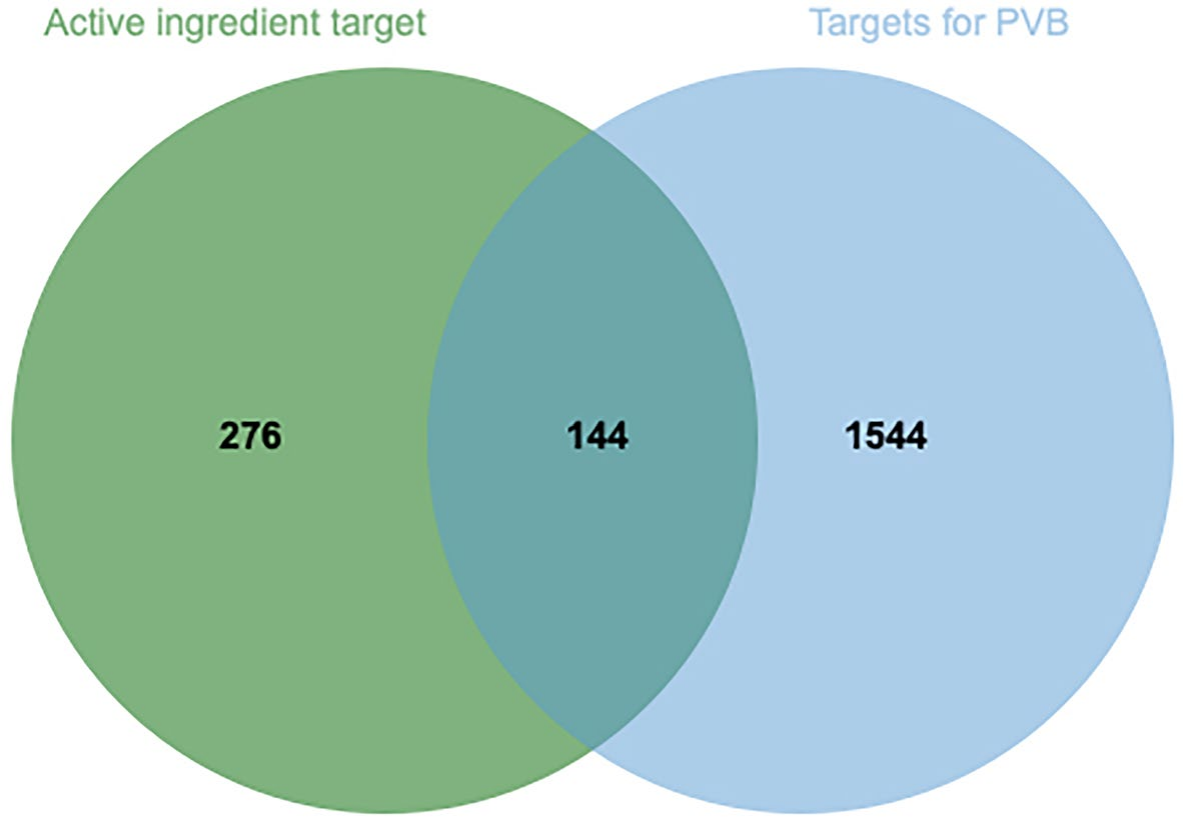
The intersecting targets of the NRER-SAL herb pair and PVB targets. The left are NRER and SAL active targets, the right are PVB targets, and the middle are intersecting targets.

A total of 5 active ingredients of NRER and 36 active ingredients of SAL were obtained by searching TCMSP databases and the literature, as shown in Table 1. The active ingredients of pine and Rhodiola rosea were named respectively. The name of NRER was GS, and the name of SAL was HJT.The total number of predicted targets of the two herbs after summary de-emphasis was 420. The four disease databases GeneCards, OMIM, DisGeNET, and Drugbank were searched to obtain 1688 PVB targets after summary deweighting. The predicted targets of NRER-SAL and the targets of PVB were plotted in a Venn diagram (see Fig. 2), and 144 intersecting targets were obtained, which were the potential targets of NRER-SAL for the treatment of PVB.

**Table 1:** NRER—SAL active ingredients.

Source (of information etc.)	Ingredients
GS1	(2R)-5,7-Dihydroxy-2-(4-hydroxyphenyl)chroman-4-one [(R)-Naringenin]
GS2	Acaciin
GS3	Acacetin
GS4	Sitosterol
GS5	Cryptotanshinone
HJT1	Sophocarpine
HJT2	Salidroside
HJT3	Acaciin
HJT4	Acacetin
HJT5	Sitosterol
HJT6	Cryptotanshinone
HJT7	2-Furanol, 5-Ethenyltetrahydro-5-methyl-2-(1-methylethyl)
HJT8	3-(4-Hydroxyphenyl)-4H-chromen-4-one
HJT9	3,5-Dihydroxy-7,3',4'-trimethoxyflavone
HJT10	3-MethvI-2-butenal
HJT11	3-Octanol
HJT12	4-Hydroxbutanoic acid
HJT13	Velutin
HJT14	Terpineol
HJT15	Benzyl beta-d-glucopyranoside
HJT16	Caffeic acid
HJT17	Phenethyl alcohol
HJT18	(1R)-()-Myrtenol
HJT19	Vanillic acid
HJT20	Tricin
HJT21	Tamarixetin
HJT22	Sabinene hydrate
HJT23	Quercetin
HJT24	Protocatechuic acid
HJT25	Phloroglucinol
HJT26	Oxiranemethanol, 3-Methyl-3-(4-methyl-3-pentenyl)-
HJT27	o-Cresol
HJT28	Kaempferol
HJT29	Herbacetin
HJT30	Gallic acid
HJT31	Ethyl gallate
HJT32	Diisobutyl phthalate
HJT33	Aurantiol
HJT34	3-Methoxygallic acid
HJT35	p-Hydroxybenzoic acid
HJT36	2-Methylhept-2-en-6-one

### "Herb-active ingredient-disease-target" network construction

**Figure 3:**
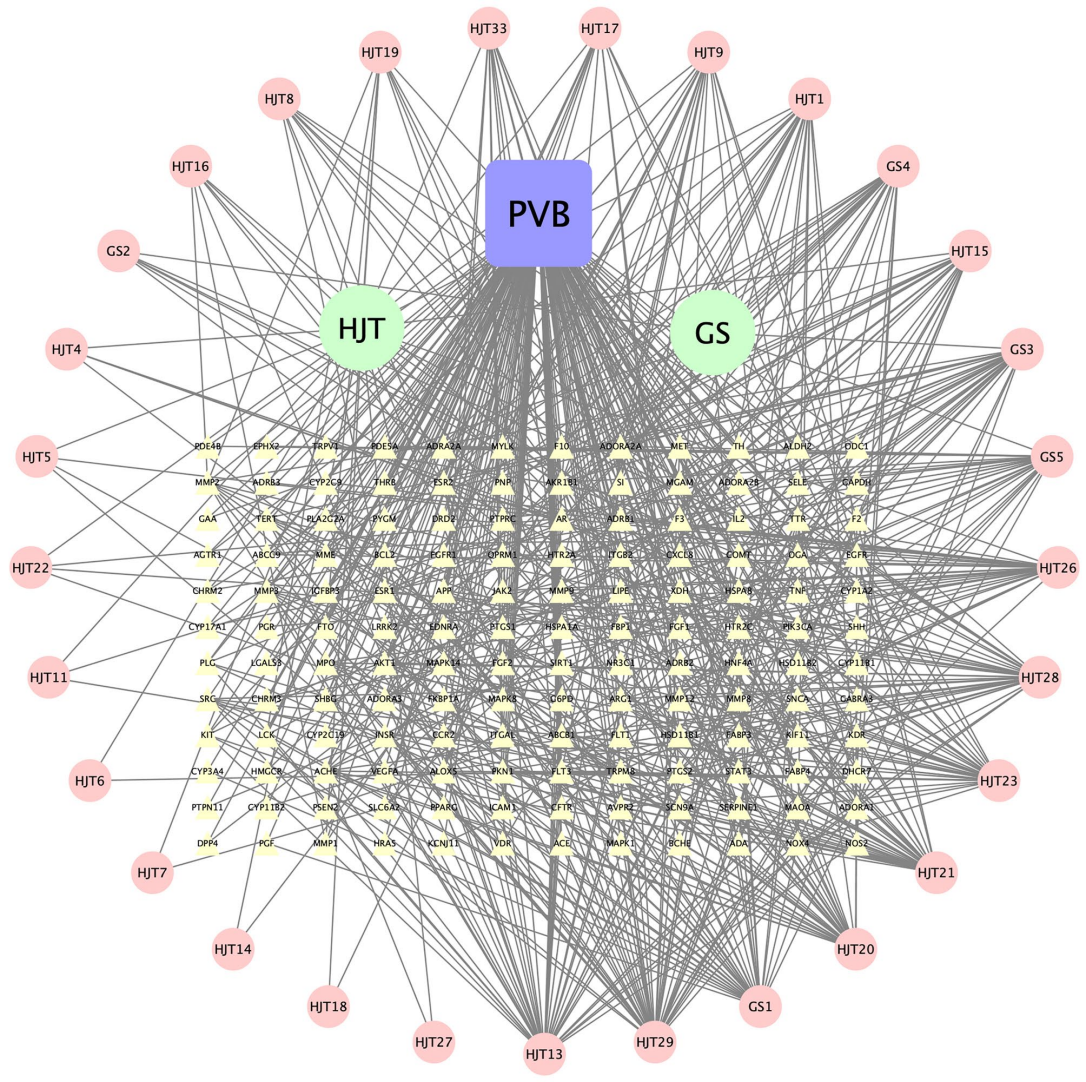
Herb-active ingredient-disease-target network diagram. Among them, the blue square represents the name of the disease, two green circles are the pinyin abbreviation of the two herbs, pink circles are the short name of the active ingredients of the two herbs (see Table 1 for details), and yellow triangles are the intersection targets of the herb and the disease, which well shows the connection between the active ingredients of NRER-SAL and PVB.

The intersecting targets were imported into Cytoscape 3.7.2 software to construct a visual network diagram of "NRER-SAL-active ingredient-PVB-targets", which is shown in Fig. 3. Among them, the green circles are the pairs of NRER and Rhodiola, the pink circles represent the active ingredients of the two herbs, and the yellow triangles indicate the intersecting targets. The network diagram clearly shows that the NRER-SAL herbs pair exerts its therapeutic effect on PVB through multiple components and targets.

### PPI network construction

**Figure 4:**
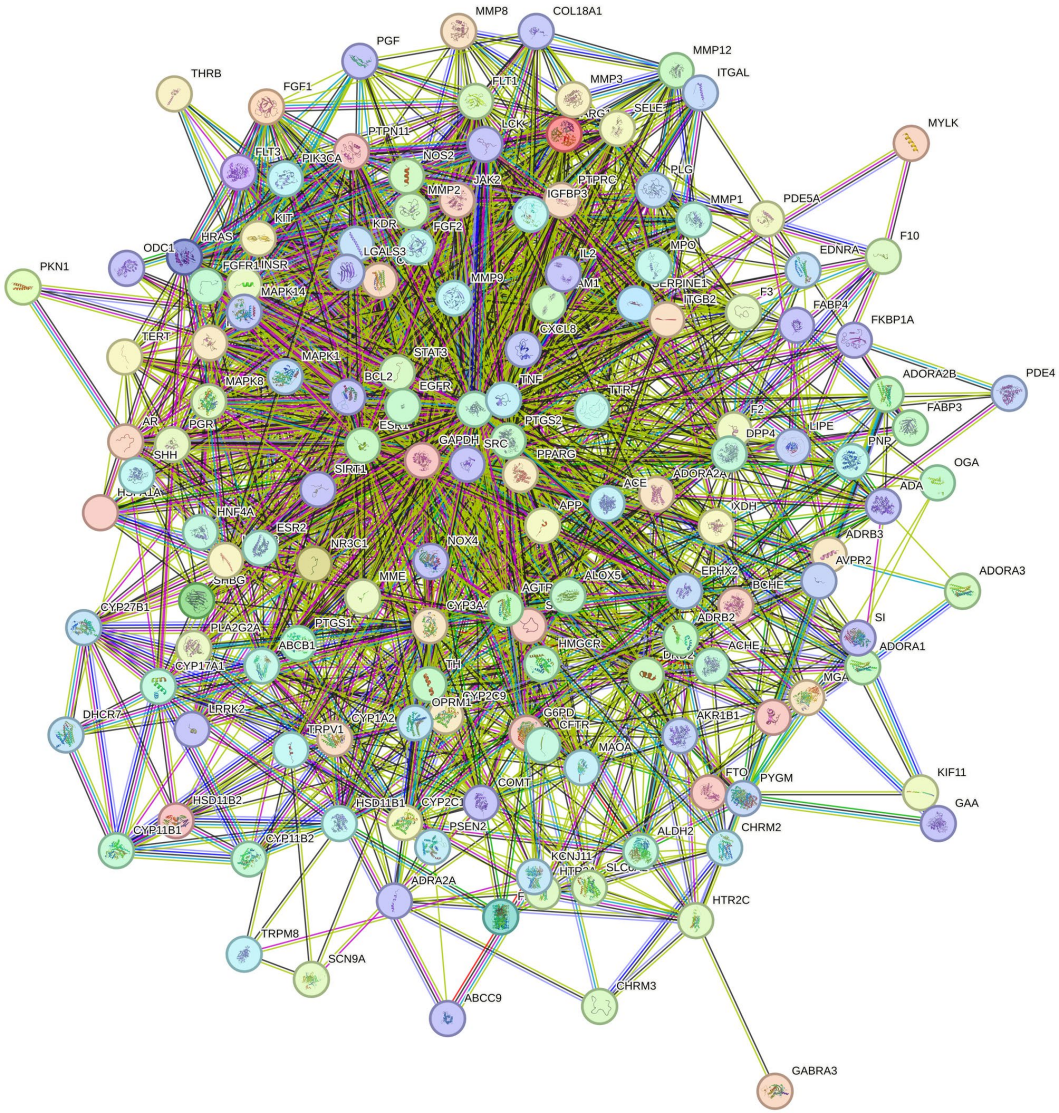
PPI network generated by STRING11.0 database. This figure generated by the STRING11.0 database, it shows the intersection relationship between intersection targets, and it shows the number of nodes are 144, the number of edges are 1875, the average node degree are 26.

**Figure 5:**
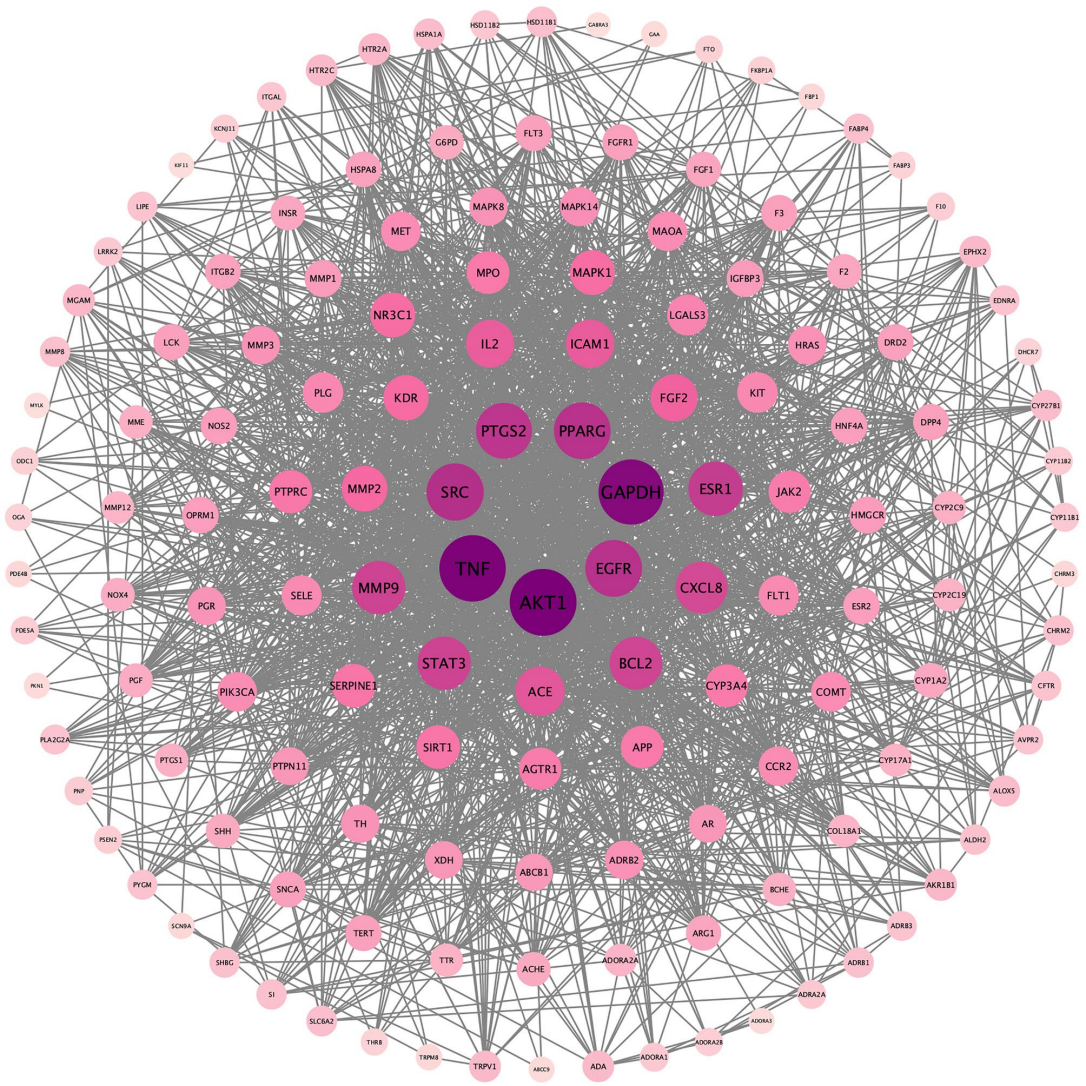
Intersection target map sorted by condition. This is the intersection target sorted by the values (Degree) from high to low at the same time. The color of the circle from purple to light pink indicates that the degree decreases, and the greater the degree value, the greater the connection degree between the gene and other genes, the greater the effect, which may be a key gene in treatment.

**Figure 6:**
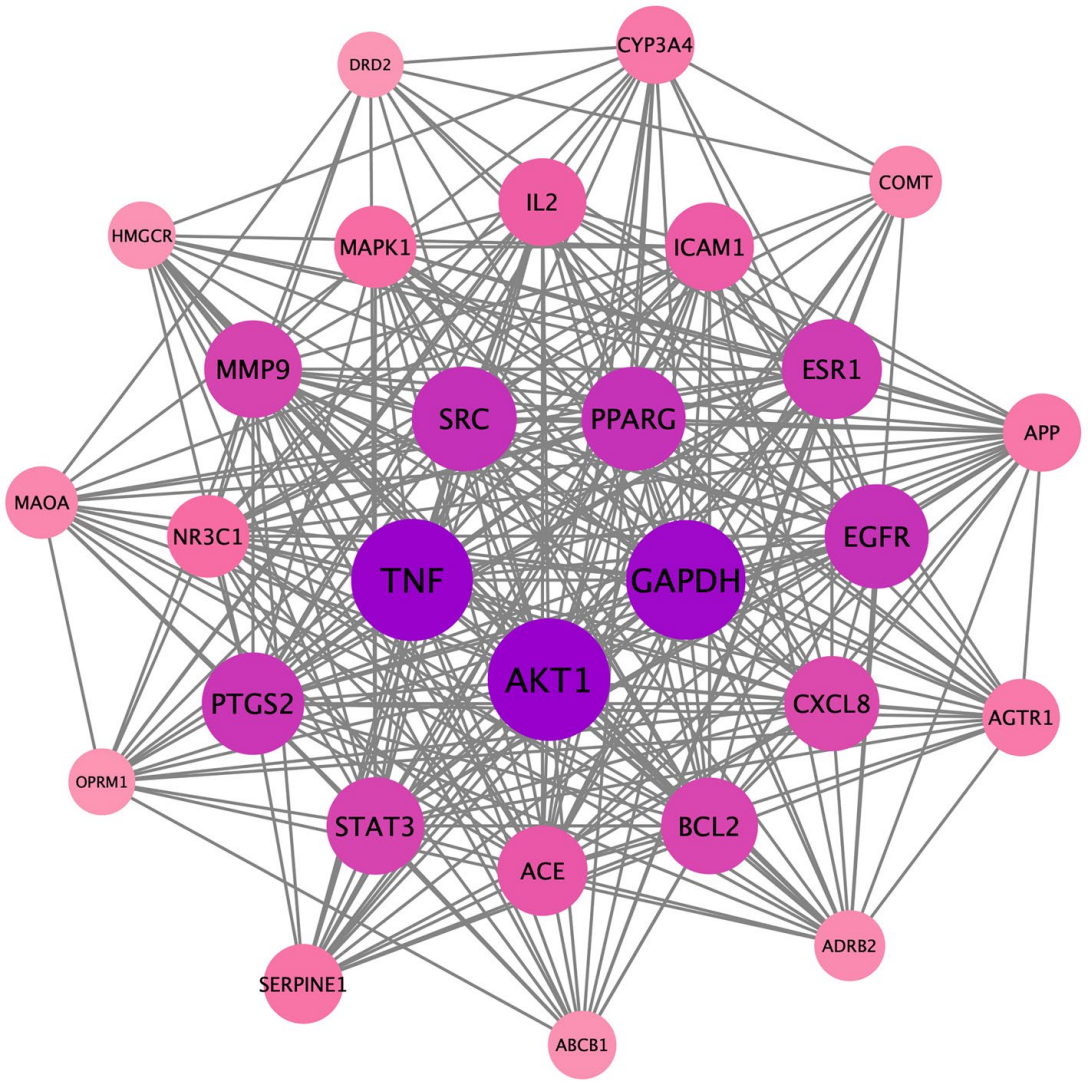
Core target network diagram. The image shows the core targets sorted by degree value from high to low after screening. Among them, the Degree ≥ 26.04, Closeness ≥ 0.0036, and Betweenness ≥ 137.56.

The PPI network was obtained through the STRING11.0 database, and the results were imported into Cytoscape3.7.2 software. This plug-in can simultaneously calculate and sort the resulting biological parameter values, and can get key nodes from the network. By default, the plug-in calculates the average value of each parameter and displays the threshold value, which is rounded to two decimal places because the calculation results loop indefinitely, and if the value is less than 1, it takes the first two digits after the decimal point^25^. The Degree ≥ 26.04, Closeness ≥ 0.0036, and Betweenness ≥ 137.56 were calculated through the plug-in CentiScape2.2, and the core targets were screened out to satisfy the above three conditions and rearranged by degree value to obtain Fig. 4. The top 10 core targets were serine/threonine protein kinase 1 (AKT1), tumor necrosis factor (TNF), glyceraldehyde triphosphate dehydrogenase (GAPDH), tyrosine kinase (SRC), epidermal growth factor receptor (EGFR), peroxisome proliferator-activated receptor gamma (PPARG), cyclooxygenase 2 (PPARG) and cyclooxygenase 2 (PPARG), cyclooxygenase 2 (PTGS2), estrogen receptor 1 (ESR1), signal transduction and transcription factor 3 (STAT3), and matrix metalloproteinase 9 (MMP9) (Figs. 5 and 6).

### GO function and KEGG pathway enrichment analysis

**Figure 7:**
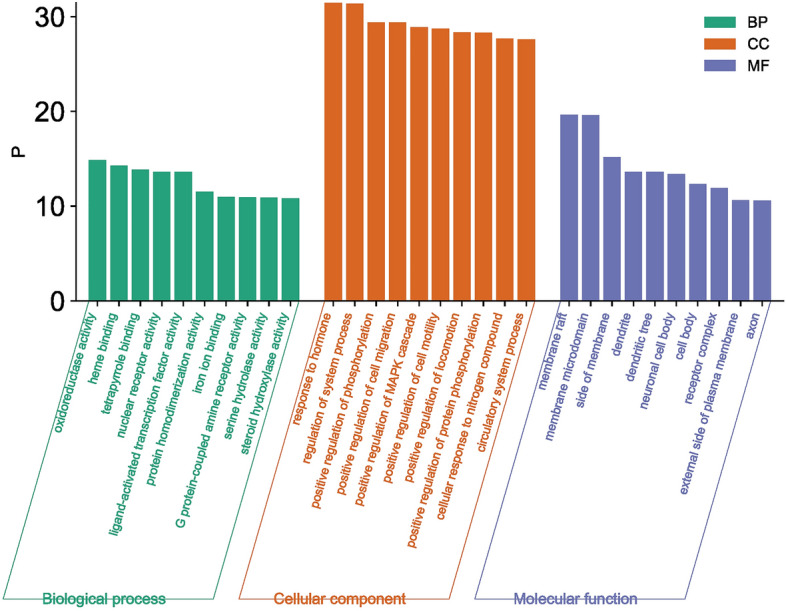
GO functional analysis histogram. BP, CC, MF are shown respectively, the horizontal coordinate represents the content of the GO analysis, the vertical coordinate represents the P-value, the green is biological process (BP), the orange is cellular component (CC), and the purple is molecular function (MF).

Through the Metascape platform GO function enrichment analysis, the molecular function (MF) mainly involves oxidoreductase activity, heme binding, tetrapyrrole binding, etc.; the biological process (BP) mainly includes response to hormone, regulation of system process and positive regulation of phosphorylation, etc.; the cellular component (CC) includes cell membrane rafts, regulation of system process and positive regulation of phosphorylation, etc. As shown in the Fig. 7 below.

**Figure 8:**
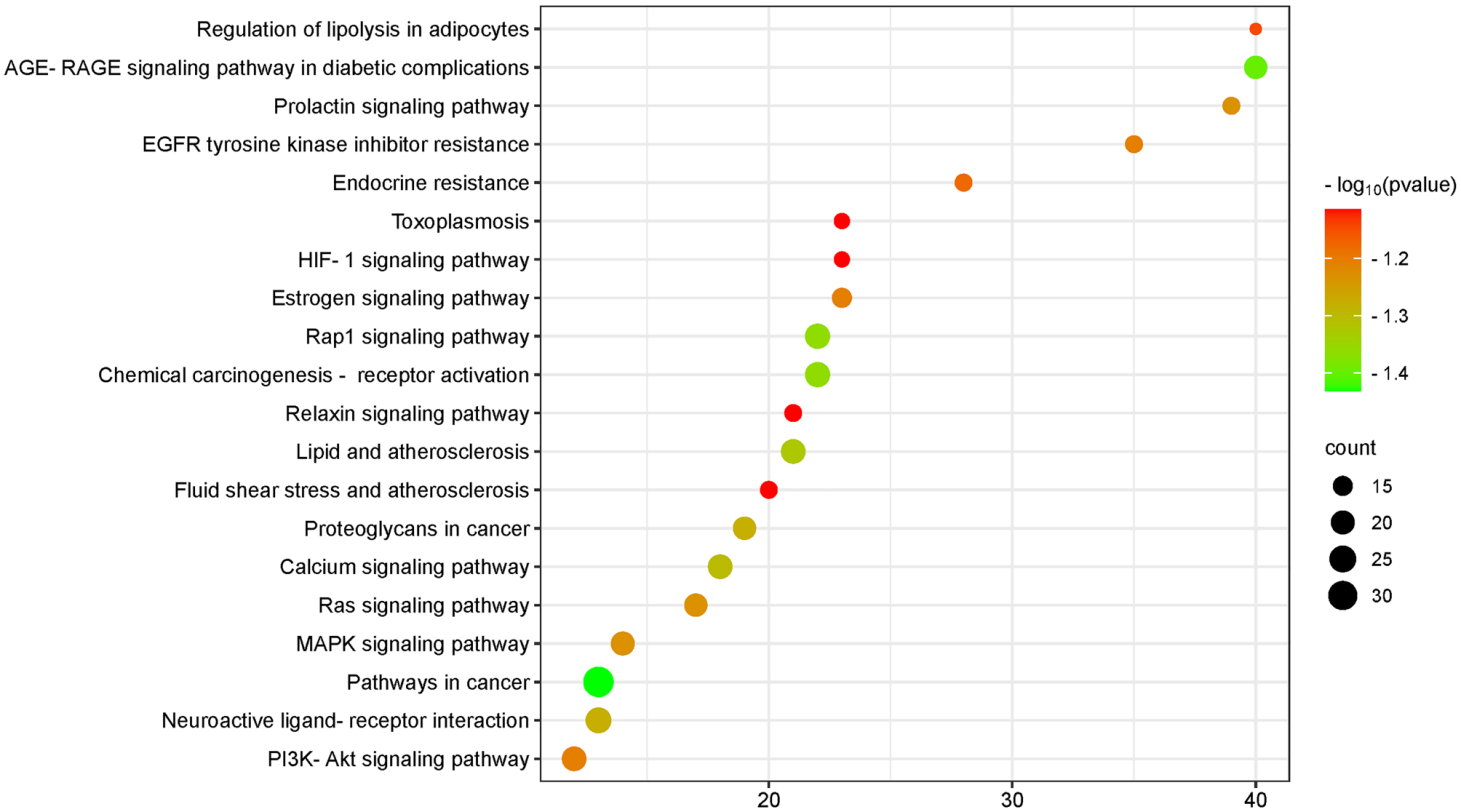
KEGG pathway bubble map. Node color varies from red to green in descending order of P-value, and node size is arranged in ascending order of number of genes.

**Figure 9:**
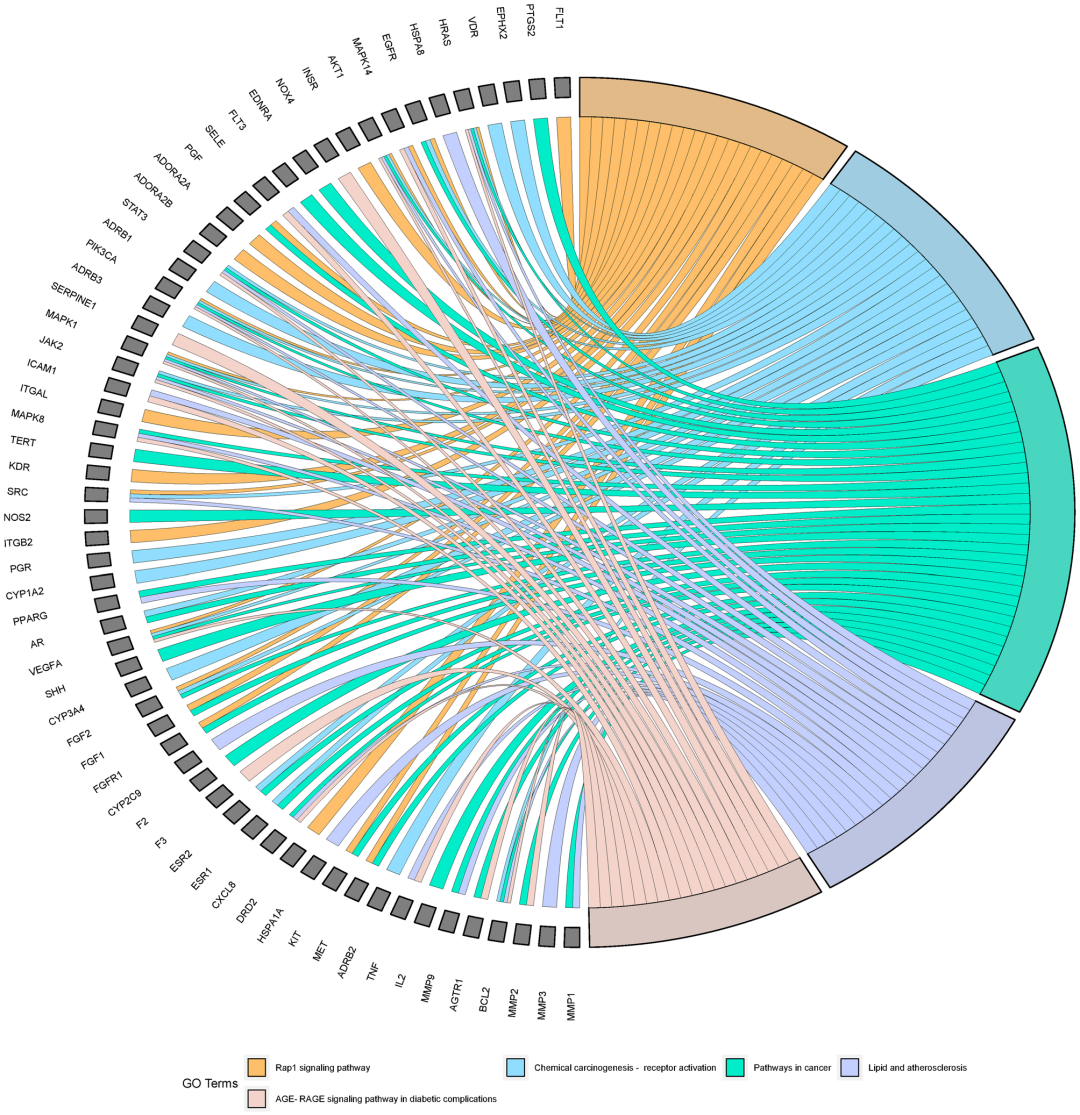
KEGG chordmap. It shows the expression of genes in different pathways. The first 5 pathways by p-valued are shown on the right and the core targets on the left for analysis, and each gene has a line connection if it is on this pathway.

KEGG-enriched pathways analysed included pathways in cancer, Calcium signaling pathway, Neuroactive ligand‒receptor interaction, Proteoglycans in cancer, Ras Proteoglycans in cancer, Ras signaling pathway, Prolactin signaling pathway, MAPK signaling pathway, Phosphatidylinositol 3-kinase/Protein kinase B signaling pathway, PI3K-Akt signaling pathway, Epidermal growth factor receptor signaling pathway (EGFR tyrosine kinase inhibitor resistance), Estrogen signaling pathway (Estrogen signaling pathway), etc., as shown below Figs. 8 and 9.

### Molecular docking

**Figure 10:**
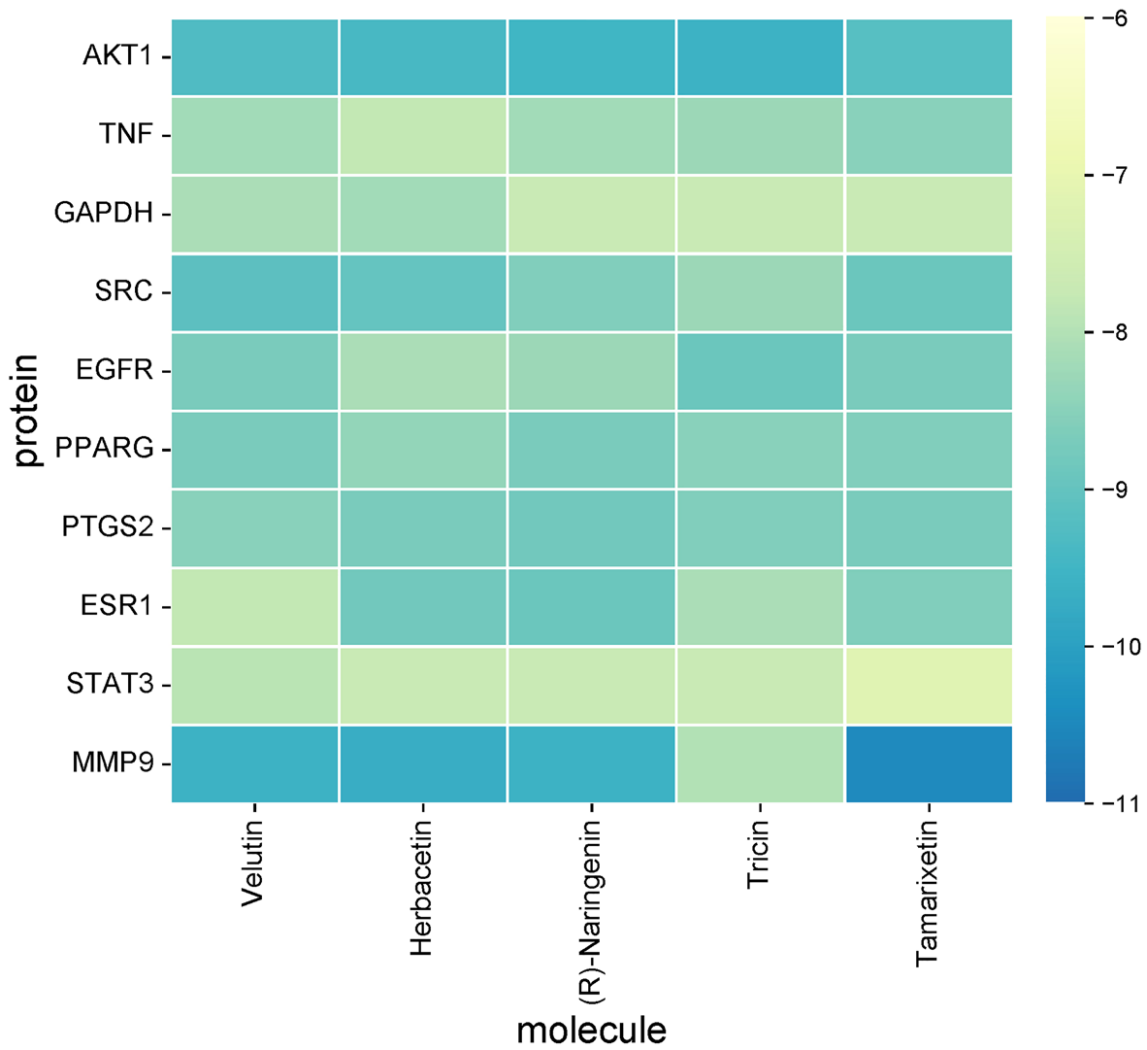
Molecular docking heatmap. The size of the binding energy is displayed by color. The darker the color, the smaller the binding energy, the better the binding.

**Figure 11:**
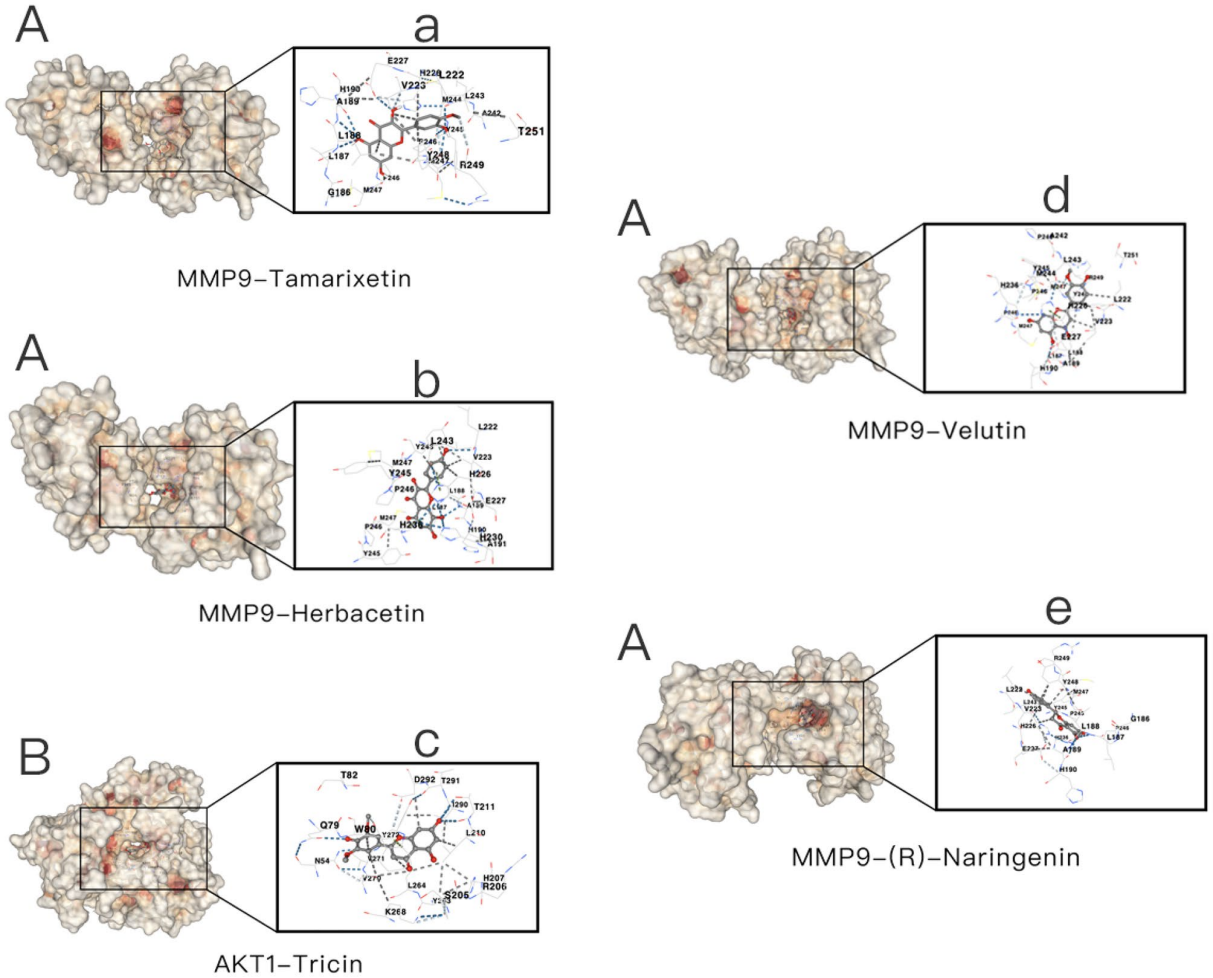
The images depict the binding positions of ligands within the protein, showcasing the interactions between the ligands and the residues. The docking mode is structure-based blind docking. The potential binding sites of the queried ligands are ranked according to AutoDock Vina score (kcal/mol), and the ones with the lowest binding energy are selected for display. A is MMP9, B is AKT1, and (**a**) is Tamarixetin, (**b**) is Herbacetin, (**c**) is Tricin, (**d**) is Velutin, and (**e**) is (R)-Naringenin.

As shown in the Fig. 10, the result shows that MMP9 communicates well with most molecules, and AKT1 communicates well with Tricin. And it can be proven that the active ingredients of the two herbs can play a modulating role on the core target. (See attached Supplementary information form1 for details) In addition, we also use heat maps (Fig. 10) to visually demonstrate the molecular docking results. Heatmap was plotted by https://www.bioinformatics.com.cn, an online platform for data analysis and visualization. It is generally believed that a binding energy < − 5 kcal/mol indicates that the protein has a better binding activity with the small molecule. The docking results of the top 5 binding energies are shown, as shown in the Fig. 11 below.

## Discussion

Accompanying the accelerated pace of life and aging, unhealthy lifestyles have led to a rising trend in the incidence of PVB, which seriously affects the quality of life and the physiological and psychological health of patients. Premature ventricular contractions.

In this study, we systematically analysed the mechanism of action of Ganoderma-Rhodiola medicinal is for the treatment of PVB by constructing a herb-PVB-related active ingredient-predictive target network and PPI and molecular docking.

NRER, contained in the "Herbal Gleanings", is mostly distributed in China's Sichuan, Gansu, Qinghai and other places. Chinese medicine believes that it has the effect of regulating qi to stop pain, open the depression to wake up the spleen, and reassure the effect of palpitations. Modern research has found that NRER has anticonvulsant epilepsy, antidepressant, anti-inflammatory, antibacterial, anti-arrhythmic and antioxidant effects and is widely used in the cardiovascular system, digestive system, endocrine system and nervous system^26,27^. The current clinically used anti-arrhythmic proprietary Chinese medicines, such as ginseng pine nutritious heart capsules and stable heart particles, all contain NRER. SAL is a genus of Rhodiola in the family Rhodiolaceae, and there are many different species, such as Rhodiola grandiflora, Rhodiola alpina, and Rhodiola sacra. Modern research has found that it can be used in the cardiovascular system. Modern research has found that it has significant therapeutic effects in cardiovascular diseases such as coronary heart disease, heart failure, myocardial infarction and arrhythmia, and it can also reduce hypoxia-induced necrosis and apoptosis of cardiomyocytes^28^. Salidroside injection, cardioplegia capsules and oral solutions of salidroside have been widely used to treat cardiovascular diseases with marked effects^29^.

Through the use of web-based pharmacological mining and organisation, it was found that the presence of a variety of components in NRER and SAL are associated with the treatment of ventricular premature expiration. It was found that^30^ SAL present in salidroside rhizome has anti-inflammatory, antioxidant and anti-apoptotic activities, which can reduce the occurrence of ventricular arrhythmia by improving myocardial ischemia and reperfusion. It was also hypothesised that Sal can affect the protein expression and phosphorylation level of myocardial Connexin43 (Cx43), correct the distribution disorder of Cx43, restore the continuity and uniformity of myocardial conduction under physiological conditions, and thus play a role in anti-ventricular premature expiration. Restoring the continuity and uniformity of myocardial conduction in the physiological state, thus exerting anti-ventricular arrhythmia effects^31–33^. Tamarixetin not only reduces the secretion of the proinflammatory cytokines interleukin 6 (IL-6), tumor necrosis factor alpha (TNF-alpha) and interleukin 12 (IL-12p70) and thus exerts anti-inflammatory effects^34^ but also inhibits apoptosis and the expression of fibrosis-related genes, reverses myocardial remodelling in the stress-overloaded heart^35^, and has a higher concentration in cardiac tissues after oral intake, which is considered to be a good cardiac target. cardiac targeting properties^36^. Tricin has been shown to increase erythrocyte count and haemoglobin concentration, with good protection against hypoxia-induced damage to cells; Tricin also has anti-inflammatory, anti-allergic and antitumour effects^37–40^. (R)-Naringenin is thought to modulate the voltage sensitivity of BKCa channels, accelerate the activation rate of the channel, change the channel gating characteristics and is not affected by intracellular Ca^2+^ concentration^41^. Herbacetin have potent anti-inflammatory properties by inhibiting the release of pro-inflammatory cytokines such as TNF-α and IL-1β, reducing cellular nitric oxide (NO) production, and reducing lipid droplets in the liver, altering the amount of fatty acid synthase (FAS), fatty acid beta-oxidation (β-oxidation), malic enzyme, glucose 6-phosphate dehydrogenase (G6PD) and carnitine palmitoyltransferase (CPT) in plasma and liver total cholesterol, triglycerides and free fatty acids^42^. Quercetin has the ability to affect ion channels and calcium homeostasis, inhibit cardiac fibrosis and inflammation, and modulate autophagy and apoptosis, thereby ameliorating ischaemia and reperfusion injury and producing benefits for arrhythmias^43^.

The core targets obtained through PPI network analysis mainly include AKT1, TNF, GAPDH, SRC, EGFR, etc. AKT1 is one of the AKT genes, and when AKT1 is overexpressed in the heart after short-term activation, it induces reversible cardiac hypertrophy, and when activated persistently, it not only causes generalised cardiac hypertrophy but also exacerbates cardiac dysfunction^44^, and cardiac hypertrophy can lead to cardiac arrhythmias and, in serious cases, malignant arrhythmias. Cardiac hypertrophy can lead to cardiac arrhythmia and, in severe cases, malignant arrhythmia^45,46^. Some studies have shown that^47^ TNF-α can affect calcium homeostasis to induce cardiac hypertrophy, and TNF and IL-1β can increase Ca^2+^ leakage in the sarcoplasmic reticulum, leading to delayed cellular depolarisation and arrhythmias^48^. Some scholars have suggested that TNF may be related to the induction of electrical remodelling through sympathetic overinternalisation^49^. GAPDH is known as glyceraldehyde-3-phosphate dehydrogenase (GAPDH), which is the most important enzyme in cardiovascular hypertrophy. Phosphate dehydrogenase (GAPDH), of which there are four types according to their cellular localisation, GAPC, NP-GAPDH, GAPA/B and GAPCp, has been associated with damaged mitochondria in response to oxidative stress induced by cardiac ischaemia‒reperfusion injury^50^, and damage to the mitochondria can lead to fatal ventricular arrhythmias^51^. It was found that SRC-mediated tyrosine kinase phosphorylates HCN4 channel proteins, and the long-term effect can induce changes in current density that can rescue the surface expression of D553N for normal channel function^52^. According to the data, EGFR and its ligand EGF protect the mouse heart against perfusion-induced ischemic injury^53^, and ischaemia‒reperfusion injury in rat myocardium can be attenuated by selective EGFR kinase inhibitors^54^.

KEGG enrichment showed that the main pathways of NRER-SAL for the treatment of PVB are the calcium signalling pathway, AGE/RAGE signalling pathway and PI3K-Akt signalling pathway. Normal cardiac physiological activity is generated by the sinus node to generate excitation and then downwards conduction. When the electrical activity is abnormal, the rhythm or frequency of the heart is disturbed. Calcium ions play a crucial role in myocardial contraction and diastole, and voltage-gated calcium channels (VGCCs), which exist on cardiomyocytes, are the key channels for Ca^2+^ inwards flow during cardiomyocyte excitation and are also involved in the ionic basis of the phase 2 plateau phase in ventricular myocytes. It has been shown that activation of the PI3K/Akt pathway can lead to Cx43 phosphorylation^55^, and activated Akt exerts antiarrhythmic effects and reduces arrhythmias by affecting Cx43 and thereby reducing the functional impairment of gap junctions or increasing the area of gap junctions^56^. This pathway also affects the occurrence of cardiac action potentials by influencing ion channels (Ikr and ICAa)^57^. It has been shown^58^ that complex inflammatory processes contribute to myocardial conduction inhomogeneity by affecting inhomogeneous changes in Na^+^ channels and cellular coupling.

Most small molecule binding occurs in protein pockets or cavities, so discovering protein–ligand binding sites and conformations is particularly important in drug discovery. Blind docking is a powerful way to obtain this information, and the results can be used for future novel drug discovery and research^59^. The minimum binding energy is the minimum energy required for the binding of molecules and proteins, which can evaluate the quality of the binding ability between molecules. The negative binding energy indicates that the two do not need to consume energy when combined, but also release energy, the structure is more stable, and the more likely to combine when they meet. The molecular docking results showed that the active ingredients of NRER-SAL have good affinity with the core targets MMP9, AKT1, TNF, GAPDH, SRC, ESR1, and EGFR, etc. which further validated the therapeutic effects of the two herbs on PVB.

In this study, we investigated the mechanism of action of NRER-SAL herbs pairing for the treatment of PVB through network pharmacological methods and concluded that it can be treated through multiple targets and pathways. The current results can provide a reference direction and guidance basis for subsequent experiments and clinics.

### Supplementary Information

Supplementary Information.

## Data Availability

All data generated or analysed during this study are included in this article.
